# Absence of the complement regulatory molecule CD59a leads to exacerbated neuropathology after traumatic brain injury in mice

**DOI:** 10.1186/1742-2094-6-2

**Published:** 2009-01-08

**Authors:** Philip F Stahel, Michael A Flierl, B Paul Morgan, Ivonne Persigehl, Christiane Stoll, Claudia Conrad, Basel M Touban, Wade R Smith, Kathryn Beauchamp, Oliver I Schmidt, Wolfgang Ertel, Iris Leinhase

**Affiliations:** 1 Department of Orthopedic Surgery, Denver Health Medical Center, University of Colorado School of Medicine, Denver, CO 80204, USA; 2 Department of Medical Biochemistry and Immunology, School of Medicine, Cardiff University, Cardiff CF14 4XN, UK; 3 Department of Trauma and Reconstructive Surgery, Charité University Medical Center, Campus Benjamin Franklin, D-12207 Berlin, Germany; 4 Department of Surgery, Division of Neurosurgery, Denver Health Medical Center, University of Colorado School of Medicine, Denver, CO 80204, USA; 5 Trauma Center St. Georg, Department of Trauma Surgery and Orthopaedics, D-04129 Leipzig, Germany

## Abstract

**Background:**

Complement represents a crucial mediator of neuroinflammation and neurodegeneration after traumatic brain injury. The role of the terminal complement activation pathway, leading to generation of the membrane attack complex (MAC), has not been thoroughly investigated. CD59 is the major regulator of MAC formation and represents an essential protector from homologous cell injury after complement activation in the injured brain.

**Methods:**

Mice deleted in the *Cd59a *gene (CD59a^-/-^) and wild-type littermates (*n *= 60) were subjected to focal closed head injury. Sham-operated (*n *= 60) and normal untreated mice (*n *= 14) served as negative controls. The posttraumatic neurological impairment was assessed for up to one week after trauma, using a standardized *Neurological Severity Score *(NSS). The extent of neuronal cell death was determined by serum levels of neuron-specific enolase (NSE) and by staining of brain tissue sections in TUNEL technique. The expression profiles of pro-apoptotic (Fas, FasL, Bax) and anti-apoptotic (Bcl-2) mediators were determined at the gene and protein level by real-time RT-PCR and Western blot, respectively.

**Results:**

Clinically, the brain-injured CD59a^-/- ^mice showed a significantly impaired neurological outcome within 7 days, as determined by a higher NSS, compared to wild-type controls. The NSE serum levels, an indirect marker of neuronal cell death, were significantly elevated in CD59a^-/- ^mice at 4 h and 24 h after trauma, compared to wild-type littermates. At the tissue level, increased neuronal cell death and brain tissue destruction was detected by TUNEL histochemistry in CD59a^-/- ^mice within 24 hours to 7 days after head trauma. The analysis of brain homogenates for potential mediators and regulators of cell death other than the complement MAC (Fas, FasL, Bax, Bcl-2) revealed no difference in gene expression and protein levels between CD59a^-/- ^and wild-type mice.

**Conclusion:**

These data emphasize an important role of CD59 in mediating protection from secondary neuronal cell death and further underscore the key role of the terminal complement pathway in the pathophysiology of traumatic brain injury. The exact mechanisms of complement MAC-induced secondary neuronal cell death after head injury require further investigation.

## Background

Clinical and experimental studies have implied a pivotal role for the membrane attack complex (MAC, C5b-9) of the terminal complement activation pathway in the pathogenesis of secondary neuronal cell death after traumatic brain injury [1-4]. The complement regulatory molecule CD59 represents the major controller of MAC formation and an essential protector of homologous cell injury after complement activation [5,6]. Neurons express CD59 constitutively to protect from autologous "innocent bystander" cell lysis after activation of the complement system in the injured brain [2,7]. However, due to low levels of neuronal CD59 expression, the neuronal capacity of controlling complement activation is very limited [7], which renders neurons susceptible to complement-mediated lysis by the MAC in the setting of intracerebral complement activation [8-10].

One of the putative mechanisms of complement-mediated neuronal death is reflected by the notion that the activation of phosphatidyl-inositol-specific phospholipase C (PI-PLC) after traumatic brain injury [11] renders neurons vulnerable to MAC-mediated lysis by shedding of the glycosyl-phosphatidyl-inositol (GPI)-anchored glycoprotein CD59 from neuronal membranes [2]. The intracerebral formation and deposition of MAC on neurons in the contusion area and penumbra zone has been shown to occur after human head injury [3,12]. However, the biological significance of CD59 in protecting from complement-mediated neuropathology after traumatic brain injury is far from being fully understood.

The present study was designed to investigate the role of CD59a in a standardized experimental model of closed head injury in mice lacking the gene for *Cd59a *(CD59a^-/-^). In mice, the *Cd59 *gene is duplicated, yielding *Cd59a *(widely expressed) and *Cd59b *(testis-restricted) [13,14]. The CD59a^-/- ^mice were previously shown to be highly susceptible to complement-mediated demyelination and axonal injury in a model of experimental allergic encephalomyelitis (EAE) [15], and thus provide an excellent *in vivo *model to investigate the role of complement-mediated membrane attack and CD59-dependent neuroprotection in the setting of traumatic brain injury. We hypothesized that CD59a^-/- ^mice would be more susceptible to complement-mediated secondary brain injury than wild-type littermates in a standardized model of closed head injury.

## Materials and methods

### Animals

The generation and characterization of CD59a^-/- ^mice was previously described [16]. These mice were found to have a spontaneous intravascular hemolysis due to erythrocyte susceptibility to complement-mediated lysis. Despite the chronic hemolysis, the CD59a^-/- ^mice are healthy and fertile, not anemic, but display elevated reticulocyte counts as a indicator of increased erythrocyte turnover [16]. The CD59a^-/- ^mice were generated on a mixed 129/Sv × C57BL/6 genetic background. Wild-type littermates of the 129/Sv × C57BL/6 strain were used as controls. All mice were of age 10–12 weeks, weighing 28–32 g, and of male gender exclusively, in order to avoid a bias regarding gender-related susceptibilty to brain injury. Animals were kept in single cages, bred in a selective pathogen-free (SPF) environment under standardized conditions of temperature (21°C), humidity (60%), light and dark cycles (12:12 h), with food and water provided *ad libitum*. A total of *n *= 134 animals were used for this study (*n *= 67 wild-type; *n *= 67 CD59a^-/-^). All experimental procedures were performed in compliance with the standards of the *Federation of European Laboratory Animal Science Association *(FELASA) and were approved by the institutional animal care committee (*Landesamt für Arbeitsschutz, Gesundheitsschutz und technische Sicherheit Berlin*, Berlin, Germany; approval No. G0308/04).

### Surgical procedures

Mice were subjected to experimental closed head injury using a standardized weight-drop device, as previously described [17]. In brief, after induction of isoflurane anesthesia, the skull was exposed by a longitudinal midline scalp incision. The head was fixed and a 250 g weight was dropped on the skull, inducing a focal blunt injury to the left hemisphere from a mean height of 1.7 ± 0.51 cm (mean ± SEM) for CD59a^-/-^, and 2.6 ± 0.48 cm for wild-type mice, respectively. The difference in selected weight-drop heights between the knockout mice and wild-type littermates is due to the observed increased susceptibility to head injury in the CD59a^-/- ^mice, leading to sudden posttraumatic death in initial validation experiments. We therefore titrated the falling height to achieve a similar extent of neurological impairment 1 h after trauma between the groups, based on the baseline posttraumatic NSS (see below). The 1 h NSS reflects the initial extent of head injury. Thus, the reduced weight-drop height in the knockout mice is an indirect sign of increased susceptibility to trauma in the CD59a^-/- ^group, independent of the additional parameters assessed at later time-points. The stratification of cohorts according to the initial NSS at 1 h has been previously described in this model system as a feasible strategy to attain comparable injury severity between the groups at baseline. This notion is supported by recent studies in the same experimental system which demonstrated a correlation between injury severity determined on MRI with the NSS at 1 hour after trauma [18,19].

After trauma, all mice received supporting oxygenation with 100% O_2 _until fully awake. They were brought back to their cages and monitored at regular time intervals for up to 7 days. Posttraumatic analgesia was provided by injection of 0.1 mg/Kg buprenorphin s.c. immediately prior to the experimental procedure. The posttraumatic neurological impairment was assessed at defined time intervals after trauma (*t *= 1 h, 4 h, 24 h, and 7 days) using a standardized *Neurological Severity Score *(NSS), as described below. Sham-operated mice underwent identical procedures with regard to anesthesia, analgesia, and surgical scalp incision, but were not subjected to experimental head trauma. Normal mice were used as an additional internal negative control group and were kept under identical conditions as the trauma and sham-operated mice, but no anesthesia, analgesia, surgical and experimental procedures were performed.

### Sample harvesting procedures

Ten mice per group and time-point were euthanized by decapitation under isoflurane anesthesia at *t *= 4 h, 24 h, and 7 days. Brains were immediately surgically removed, divided into left (injured) and right (contralateral/uninjured) hemispheres, snap-frozen in liquid nitrogen and stored at -80°C until analysis. In addition, serum samples were collected prior to decapitation by intracardiac puncture at identical time-points for determination of neuronal cell death markers by ELISA. Serum samples were collected in sterile tubes, centrifuged at 12,000 rpm at 4°C for 20 min, aliquoted and frozen at -80°C until analyzed.

### Neurological severity score (NSS)

A previously characterized 10-parameter score was used for assessment of posttraumatic neurological impairment, as described elsewhere in detail [20]. The NSS was assessed in a blinded fashion by two different investigators at the time-points *t *= 1 h, 4 h, 24 h, and 7 days after trauma. The baseline NSS at 1 hour reflects the severity of the initial injury. The score comprises 10 individual parameters, including tasks on motor function, alertness, and physiological behavior, whereby one point is given for failure of the task, and no point for succeeding. A maximum NSS score of 10 points indicates severe neurological dysfunction, with failure of all tasks. A spontaneous recovery over time, for up to 4 weeks after trauma, is observed in this model system, as previously described [17,20,21].

### Quantification of neuron-specific enolase

Serum levels of neuron-specific enolase (NSE), an established marker of neuronal cell death after head injury [22], were determined by a commercially available ELISA, specific for human NSE (Immuno-Biological Laboratories, Minneapolis, MN). Following confirmation of cross-reactivity of mouse NSE, samples were diluted 1:10 and analyzed by ELISA according to the manufacturer's protocol. Absorbance was read at 450 nm using a "SpectraMax 190" reader (Molecular Devices, Sunnyvale, CA). All samples were analyzed in duplicate and results were calculated from the means of duplicate sample analysis. The sensitivity of the assay was 1 pg/ml, and the standard curve was linear from 1 pg/ml to 140 pg/ml.

### Assessment of neuronal cell death

The terminal deoxynucleotidyl transferase dUTP nick-end labeling (TUNEL) technique was applied, using the "Fluorescein *In Situ *Cell Death Detection Kit" (Roche Diagnostics GmbH, Mannheim, Germany), according to the manufacturer's instructions, to determine the extent of neuronal cell death in tissue sections, as previously described [23,24]. In brief, slides were dried for 30 min followed by fixation in 10% formalin solution at RT. After washing in PBS, sections were incubated in ice-cold ethanol-acetic acid solution (3:1), washed in PBS and incubated with 3% Triton X-100 solution for 60 min at RT for permeabilization. Slides were then incubated with the TdT-enzyme in reaction buffer containing fluorescein-dUTP for 90 min at 37°C. Negative control was performed using only the reaction buffer without TdT enzyme. Positive controls were performed by digesting with 500 U/ml DNase grade I solution (Roche). To preserve cells for comparison, slices were covered with Vectashield^® ^mounting medium containing 4',6'-diamino-2-phenylindole (DAPI; Vector). All samples were evaluated immediately after staining using an "Axioskop 40" fluorescence microscope (Zeiss, Germany) at 460 nm for DAPI and 520 nm for TUNEL fluorescence. Data were analyzed by Alpha digi doc 1201 software (Alpha Innotech, San Leandro, CA).

### Real-time RT-PCR

Changes in the mRNA expression profiles of pro-apoptotic (Fas, FasL, Bax) and anti-apoptotic (Bcl-2) mediators were determined by semi-quantitative two-step real-time RT-PCR using commercially available, murine-specific primers (Qiagen, Hilden, Germany). The amplicon lengths were 136 bp for GAPDH (Qiagen No. 241012), 96 bp for Fas (Qiagen No. 241122), 109 bp for FasL (Qiagen No. 241194), 146 bp for Bax (Qiagen No. 241116), and 118 bp for Bcl-2 (Qiagen No. 241118). The detailed technique used in our laboratory was previously described [24,25]. In brief, the left brain hemispheres were homogenized as individual hemispheres in Qiazol^® ^buffer (Qiagen). Total RNA was isolated and further purified using RNeasy^® ^Mini-kits (Qiagen) and RNA concentrations were measured using a spectrophotometer (Bio-Rad, Munich, Germany). From each brain hemisphere, 2 μg RNA were reversely transcribed using random nonamer and oligo-dT16mer primers (Operon Biotechnologies, Cologne, Germany) with Omniscript^® ^kits (Qiagen), according to the manufacturer's instructions. Real-time RT-PCR was performed using validated commercially available and custom designed primer-probe^® ^sets (Qiagen) and optimized protocols on the Opticon^® ^real-time PCR Detection System (Bio-Rad). For quantification of gene expression levels, GAPDH amplicons were generated and used as a "house-keeping" internal control gene. Relative gene expression levels were calculated in relation to the corresponding GAPDH gene expression levels.

### Western blotting

The protein levels of pro- and anti-apoptotic mediators were determined in homogenized mouse brains by Western blot analysis, as previously described [23,25]. Briefly, mouse brains were surgically removed under anesthesia, separated into left and right hemispheres, and immediately homogenized in lysis buffer (Sigma) containing 100 mM TRIS-HCl (pH 7.5), 150 mM NaCl, 0.5% sodium dodecyl sulfate (SDS), 0.5% Nonidet P-40, 10 μg/ml aprotinin, 10 μg/ml leupeptin, 5 μg/ml pepstatin, 1 mM phenyl-methyl-sulfonyl fluoride in deionized water, using an Ultra Turrax Homogenizer^® ^(IKA Werke, Staufen, Germany). After 15 min centrifugation at 13,000 × g, the protein content of the supernatants was determined by commercially available colorimetric protein assay ("BCA Protein Assay", Pierce/Perbio Science, Bonn, Germany). A 60 μg sample of total protein was denatured in loading buffer and separated under reducing conditions on 10% (for Fas, FasL) or 12% (for Bax, Bcl-2) SDS-polyacrylamide gels in parallel with a broad range prestained SDS-PAGE protein standard (Bio-Rad, Munich, Germany). Proteins were then transferred to Protean BA 83 nitrocellulose membranes (Schleicher & Schuell, Dassel, Germany) by electroblotting (Bio-Rad). The blots were blocked overnight and then incubated with either polyclonal rabbit anti-mouse Fas (1:200), rabbit anti-mouse FasL (1:200), rabbit anti-mouse Bax (1:300), or monoclonal anti-mouse Bcl-2 (1:500) antibodies (Santa Cruz Biotechnology, Heidelberg, Germany), and with a monocloncal anti-β-actin antibody (clone AC-15, Sigma) diluted 1:10,000, as internal control for ascertaining equal loading of the bands. After incubation with HRP-labelled secondary antibodies (Dako, Hamburg, Germany, and Santa Cruz Biotechnology, Heidelberg, Germany), diluted 1:5,000, antibody binding was visualized by a non-radioactive chemiluminescence technique using a commercially available ECL^® ^Western blotting kit (Amersham Pharmacia Biotech, Freiburg, Germany). Equal transfer of proteins to the blotting membrane was confirmed by ponceau red staining (Sigma).

### Statistical analysis

Statistical analysis was performed using commercially available software (SPSS 9.0 for Windows™). Differences in intracerebral gene expression levels and NSE serum levels between the groups were determined by the unpaired Student's *t*-test. The repeated measures analysis of variance (ANOVA) was used for assessing differences in neurological scores (NSS). A *P*-value < 0.05 was considered statistically significant.

## Results

### Neurological outcome

**Figure 1:**
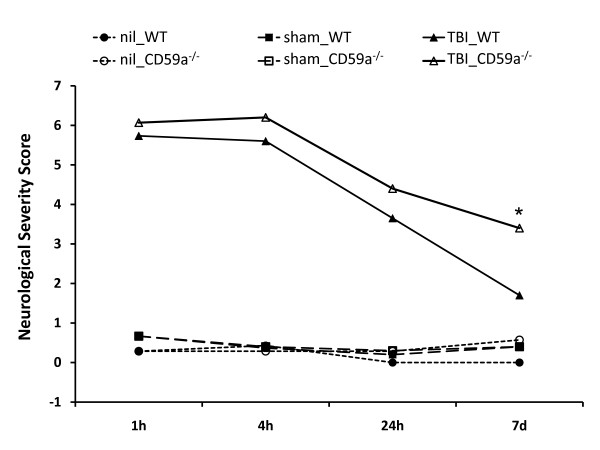
**Neurological outcome after head injury in wild-type and CD59^-/- ^mice**. The posttraumatic neurological impairment was determined by a standardized 10-parameter *"Neurological Severity Score" *(NSS) in normal untreated ("nil"), sham-operated, and head-injured mice from 1 h to 7 days after trauma (total: *n *= 134 mice). The neurological assessment was performed in a blinded fashion by two different investigators. A maximal score of 10 points corresponds to a severe neurological impairment, while a score of 0 points reflects normal behavior. The graph shows the resultant median levels of the groups at different time-points. Normal untreated mice (*n *= 7 per group) and sham-operated mice (*n *= 30 per group) had normal neurological scores ranging from 0–2 points, with no difference between wild-type and CD59a^-/- ^mice. In head-injured mice (*n *= 30 per group), the baseline NSS was titrated to be in a similar range between head-injured and CD59a^-/- ^mice at 1 h (*P*> 0.05). By 7 days after trauma, the CD59a^-/- ^mice had a significantly worse outcome compared to wild-type littermates, as reflected by a significantly increased NSS (*P < 0.05, repeated measures ANOVA). TBI, traumatic brain injury; WT, wild-type.

The assessment of of neurological tasks was performed by two investigators who were blinded about the treatment groups (Figure 1). Normal untreated mice ("nil") and sham-operated control mice displayed a normal behavior, with a range of NSS scores between 0–2 points in all control groups. In contrast, head-injured mice in both treatment groups had a siginifcantly increased NSS at all time-points assessed for up to 7 days after trauma, compared to the control groups (*P *< 0.05, repeated measures ANOVA; Figure 1). In the trauma cohorts, the baseline NSS at 1 h was titrated by adjusting the falling height of the weight-drop device (see methods section), in order to achieve a similar range of initial injury severity between wild-type (median levels 5.7) and CD59a^-/- ^mice (median levels 6.1). A spontaneous, physiological neurological recovery was seen in both treatment groups over time, as reflected by a decreased NSS at 7 days compared to 1 h after TBI. By 7 days, the head-injured wild-type mice showed a significantly decreased NSS, compared to the knockout animals, implying a more severe neurological impairment in the absence of CD59a at one week after trauma (median levels 1.7 in wild-type *vs. *3.4 in CD59a^-/- ^mice; *P *< 0.05; Figure 1).

### NSE serum levels

**Figure 2:**
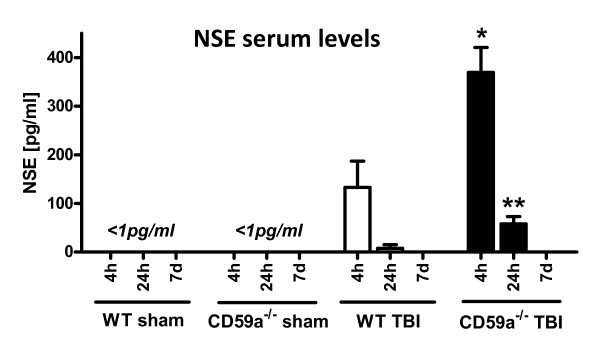
**Serum levels of neuron-specific enolase (NSE), a marker of neuronal cell death**. NSE protein levels were quantified in serum samples by ELISA, as described in the methods section. In sham-operated wild-type and CD59a^-/- ^mice, NSE was not detectable in any sample at any time-point, i.e. below the sensitivity of the assay at 1 pg/ml. In contrast, NSE serum levels were significantly elevated at 4 h and 24 h after trauma, both in wild-type and CD59a^-/- ^mice, compared to sham-operated controls. In addition, the NSE serum levels were significantly higher at both time-points in head-injured CD59a^-/- ^mice, compared to wild-type littermates (*P < 0.05 for *t *= 4 h, **P < 0.05 for *t *= 24 h). This implies a greater extent of neuronal cell death in the genetic absence of *Cd59a*. NSE was not detectable in head-injured mice at 7 days. Data are shown as mean ± SEM for *n *= 3 per group and time-point. TBI, traumatic brain injury; WT, wild-type.

The quantification of NSE levels in serum samples, as a standard marker of neuronal injury [22], revealed non-detectable levels (below the sensitivity of the ELISA at 1 pg/ml) in sham-operated wild-type and CD59a^-/- ^mice, at all time-points assessed (Figure 2). In contrast, NSE serum levels were detectable in head-injured mice at 4 h and 24 h, but not at 7 days after trauma. In CD59a^-/- ^mice, the mean NSE levels were significantly higher at 4 h (369.5 *vs. *132.8 pg/ml) and 24 h (58.0 *vs. *7.5 pg/ml) after trauma, compared to wild-type littermates (*P *< 0.05; Figure 2).

### Neuronal cell death

**Figure 3:**
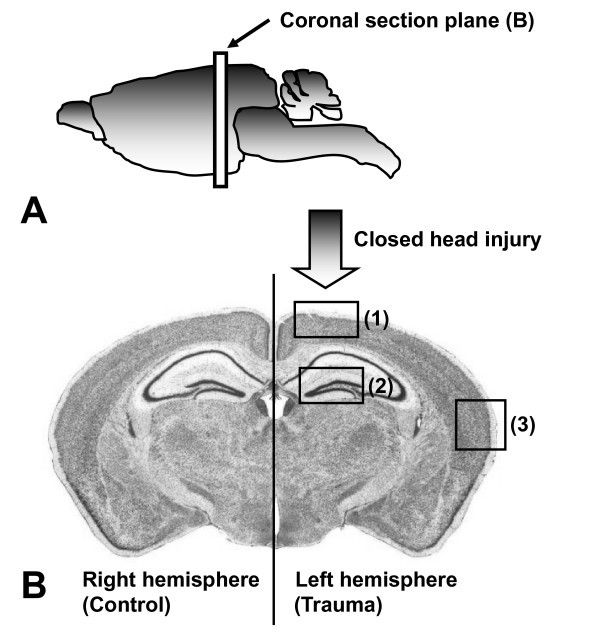
**Schematic depiction of the anatomic regions of interest in coronal brain tissue sections from injured mouse brains**. Closed head injury was applied to the left hemisphere, as described in detail in the methods section. The right (contralateral) hemisphere served as internal negative control. The anatomic regions representing the tissue sections analyzed by TUNEL histochemistry in figure 4, include: (1) Contusion site in the left hemisphere; (2) Hippocampus; (3) Remote cortex in the ipsilateral hemisphere.

**Figure 4:**
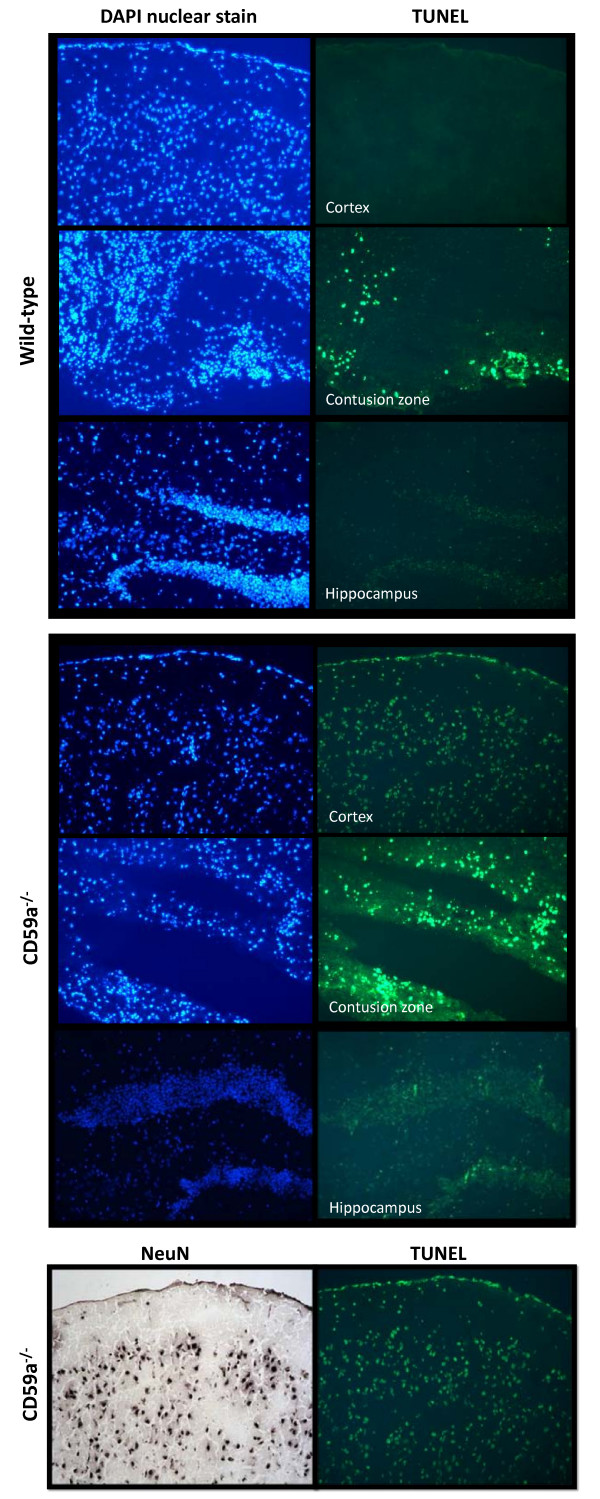
**Exacerbated brain tissue destruction and neuronal cell death in head-injured CD59^-/- ^mice**. Microphotographs show adjacent cryosections of 8 μm thickness of left (injured) brain hemispheres harvested at 24 h after closed head injury each from a representative wild-type (upper panel) and a CD59a^-/- ^(lower panel) mouse. Cellular signals were visualized by fluorescence microscopy. The left columns represent the DAPI nuclear stain which reveals the cellular distribution and morphology of all cells present in the resultant sections. TUNEL histochemistry was performed on adjacent sections (right columns) to reveal the distribution and morphology of cell death. TUNEL-positive cells were mainly detected within the contusion zone of head-injured wild-type mice. In contrast, the number of TUNEL-positive cells was clearly augmented in the contusion zone of CD59a^-/- ^mice, and remote cell death was detected in cortical layers of the ipsilateral hemisphere and in the hippocampus. Neurons were identified as the main cellular source of TUNEL-positive cell types by immunostaining of adjacent brain sections using anti-NeuN as primary antibody, a neuron-specific cell marker (bottom panels). Uninjured, i.e. sham-operated or untreated, CD59^-/- ^mice did not show any positive TUNEL signals in brain sections (data not shown). These data imply an increased amount of cell death and tissue destruction in brain-injured CD59^-/- ^mice, compared to wild-type animals. Original magnifications: 100×.

As previously described for this experimental model [24,25], head-injured wild-type mice exhibited a massive destruction of their cortical neuronal layers at the contusion site, as determined by immunohistochemistry using a specific anti-NeuN Ab as a neuronal marker. Figure 3 depicts the different anatomic regions assessed in coronal brain tissue sections. The histological investigation of intracerebral cell death by TUNEL histochemistry revealed a dramatic increase in TUNEL-positive neurons in the injured left hemispheres of wild-type mice, from 4 hours to 7 days after trauma (Figure 4, representative stainings for *t *= 24 h). In accordance with previously published findings [23,24], TUNEL-positive neurons were detected within the contusion zone (Figure 4) and the hippocampus (data not shown) of the injured hemisphere of wild-type mice for up to 7 days after trauma. Head-injured CD59a^-/- ^mice showed a massive increase in the extent of brain tissue destruction and TUNEL-positive cells around the contusion site and in the ipsilateral cortex at 24 h (Figure 4), for up to 7 days after trauma (data not shown). Sham-operated or untreated CD59^-/- ^mice did not show any positive TUNEL signals in any brain section (data not shown). The immunohistochemical staining of adjacent brain sections by specific cell markers for neurons (anti-NeuN), astrocytes (anti-GFAP), microglia and infiltrating leukocytes (anti-CD11b), revealed that neurons were the predominant TUNEL-positive cell type in all sections taken from the injured hemispheres of wild-type and CD59a^-/- ^mice. TUNEL-positive cells were furthermore confirmed as neurons by their typical cellular size, morphology, and position in typical neuronal layers (Figure 4). As previously described, TUNEL-positive cells and the extent of cortical tissue destruction were less apparent in the contralateral (right) hemisphere as compared to the injured (left) hemisphere at all time-points assessed after trauma, in both groups [23,24]. The representative microphotographs shown in Figure 3 were highly reproducible in all tissue sections and animals throughout this study.

### Mediators of apoptosis

**Figure 5:**
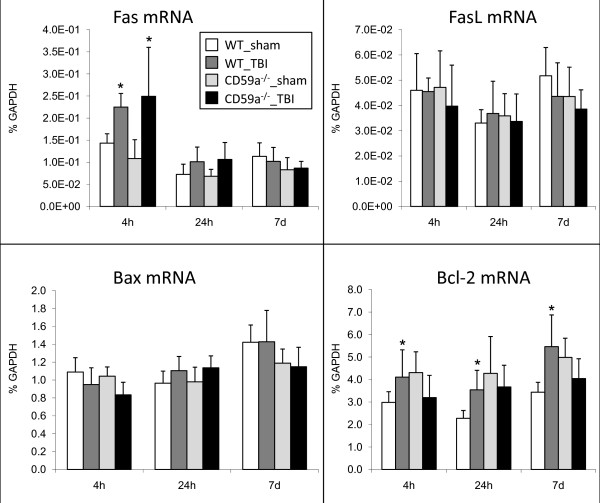
**Regulation of intracerebral gene expression for apoptotic mediators**. Total RNA was extracted from homogenized murine brains (injured left hemispheres) at 4 h, 24 h, and 7 days after sham surgery or experimental head injury. Gene expression levels of Fas, FasL, Bax and Bcl-2 were determined by semi-quantitative two-step real-time RT-PCR, as described in the methods section. A significant induction of Fas gene expression was detected at 4 h in both trauma groups, compared to sham-operated controls (*P < 0.05, unpaired Student's *t*-test). In addition, head-injured wild-type mice showed a significant induction of Bcl-2 expression at all time-points after trauma, compared to sham-operated controls (*P < 0.05). No significant differences between head-injured wild-type and CD59a^-/- ^mice were detected for any gene at any time-point. Data are shown as mean ± SEM for *n *= 6 per group and time-point. TBI, traumatic brain injury; WT, wild-type.

**Figure 6:**
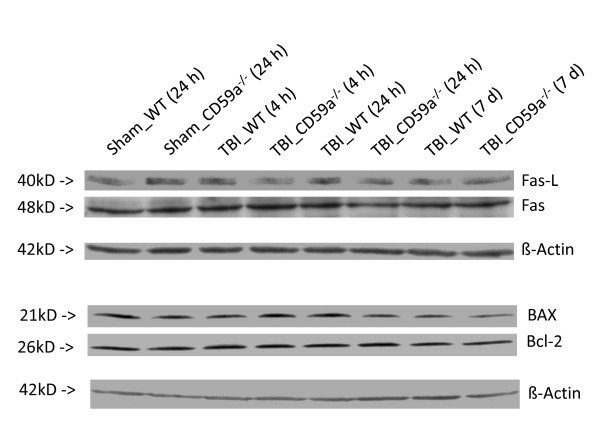
**Regulation of intracerebral protein levels of apoptotic mediators**. Western blot analysis was performed in murine brain homogenates (injured left hemispheres) using mouse-specific primary antibodies and detection by a non-radioactive chemoluminescence assay, as described in the methods section. Equal protein amounts (60 μg per lane) were loaded on SDS-PAGE and consistent loading and blotting was confirmed by ponceau staining (not shown) and control blotting with β-actin. The Western blots shown in this are representative for *n *= 3 per experiment. No significant differences were seen between the groups at a qualitative (figure) or quantitative level (not shown). TBI, traumatic brain injury; WT, wild-type.

Homogenized brain tissue samples were analyzed by real-time RT-PCR and Western blotting for gene and protein expression of Fas, FasL, Bax and Bcl-2. A significant upregulation of Fas mRNA was detected in head-injured wild-type and CD59a^-/- ^mice at *t *= 4 h, compared to sham-operated controls (*n *= 6 per group and time-point, *P *< 0.05, unpaired Student's *t*-test; Figure 5). In addition, the gene for the mitochondrial ant-apoptotic mediator Bcl-2 was significantly upregulated in injured brains of wild-type mice, compared to sham-operated controls, at all time-points assessed (*P *< 0.05; Figure 5). Head-injured CD59a^-/- ^mice showed a trend towards decreased Bcl-2 expression at all time-points after trauma, which however was not statistically significant (*P *> 0.05; Figure 5). No significant differences in Fas, FasL, Bax and Bcl-2 expression in brains of wild-type vs. CD59a^-/- ^mice were detected at any time-point for up to 7 days after trauma, either at the gene (Figure 5) or protein level (Figure 6; representative Western blot experiments). However, Bax protein expression appeared to be moderately upregulated in CD59a^-/- ^mice 4 h after trauma, when compared to wild-type littermates. The digital quantification of protein bands on Western blot membranes did not reveal any statistically significant differences between the groups at any time-point (*n *= 3 per group and time-point, *P *> 0.05; data not shown).

## Discussion

The present study was designed to evaluate the role of the complement regulatory molecule CD59a in the posttraumatic neuropathology after experimental closed head injury in mice. We sought to test the hypothesis that mice deficient in the *Cd59a *gene (CD59a^-/-^) would display a significantly increased susceptibility to complement-mediated secondary brain injury with regard to the extent of posttraumatic neurological impairment and neuronal cell death. CD59a^-/- ^mice had a significantly impaired neurological outcome 7 days after experimental closed head injury, compared to wild-type controls. This neurological deterioration occurred despite equal titration of initial severity of injury, as determined by a similar baseline NSS in wild-type and CD59a^-/- ^mice 1 h after trauma (5.7 vs. 6.1 points, mean NSS at 1 h; Figure 1). Strikingly, the titrated weight drop falling height required to induce a similar severity of injury was significantly less in the knockout mice (1.7 cm) than in wild-type mice (2.6 cm), implying an intrinsically increased baseline susceptibility to brain injury in CD59a^-/- ^mice. Aside from the clinical deterioration within 7 days, CD59a^-/- ^mice also showed a significant exacerbation of neuronal cell loss in the injured brain, as determined by (a) increased serum levels of NSE – an established marker of neuronal cell death – compared to wild-type mice (Figure 2), and (b) increased neuronal cell death and brain tissue destruction in TUNEL histochemistry experiments (Figure 4).

Since programmed cell death represents an important mechanism of secondary neuronal cell death after head injury, and since complement and the MAC have been associated with neuronal apoptosis [10,26,27], we sought to further investigate the expression of apoptotic mediators in injured brains of wild-type and CD59a^-/- ^mice. Interestingly, there was no significant difference in intracerebral expression of pro- (Fas, FasL, Bax) or anti-apoptotic mediators (Bcl-2) at the gene and protein level between CD59a^-/- ^and wild-type mice, at any time-point investigated after trauma (Figures 5 and 6). These findings suggest that the exacerbated induction of neuronal cell death in injured brains of CD59a^-/- ^mice may reflect direct MAC-mediated cellular lysis in large part, as opposed to indirect cell death mechanisms by differential regulation of apoptotic mediators. However, this notion remains speculative and requires further investigation of the detailed, molecular mechanisms of posttraumatic neuronal cell loss in head-injured CD59a^-/- ^mice.

The complement system has been implicated for more than a decade in the pathophysiology of traumatic brain injury by contributing to neuroinflammation and secondary neuronal cell death [2]. However, the exact cellular and molecular mechanisms of complement-mediated neuropathology after head injury remain far from being fully understood. Recent studies have determined that all three activation pathways (i.e. classical, alternative, and lectin) are involved in the pathophysiology of posttraumatic complement activation in the injured brain [23,24,28,29]. Interestingly, the terminal complement pathway which leads to generation of the MAC (C5b-9), also termed the "killer molecule of complement" [30], has received less attention in recent research in the field of traumatic brain injury.

We have previously shown that soluble MAC levels are significantly increased in the intrathecal compartment of patients with severe head injuries, and were associated with the extent of posttraumatic blood-brain barrier (BBB) dysfunction [3]. Other groups have shown that the MAC is a potent inducer of intracerebral neuropathology and neuroinflammation, by mediating the upregulation of adhesion molecules and leukocyte infiltration in the subarachnoid space and cerebral parenchyma within a few hours of intracerebroventricular MAC injection [31]. In addition, MAC injection into hippocampus was shown to evoke seizures and neurocytotoxicity [32]. A different study used an *in vitro *model of BBB damage and revealed that the co-incubation of normal human cerebrospinal fluid with normal serum from healthy donors resulted in complement activation and soluble MAC (sC5b-9) formation [33]. Mead *et al. *described a crucial role of the MAC in contributing to demyelination and axonal injury in studies of EAE in C6-deficient mice [9]. In a model of experimental weight-drop head injury in rats, complement C9 deposition was demonstrated around the cerebral contusion site, implying MAC deposition in injured brain tissue [34].

The complement regulatory molecule CD59 is a GPI-anchored molecule which controls MAC assembly in cellular membranes and thus protects from homologous cell lysis after complement activation [5]. CD59a has been described as the primary regulator of MAC assembly in the mouse, since mice have a testis-restricted CD59b expression [13]. We have recently described a significant induction of *Cd59a *gene upregulation in the injured mouse brains, using the same experimental closed head injury model as in the present study [25]. It appears that the upregulation of CD59a in injured brains represents a feedback mechanism aimed at protecting neurons from accidental homologous cell lysis related to posttraumatic complement activation [2]. However, in the complex setting of head injury-induced neuroinflammation, the shedding of the GPI-anchored glycoprotein CD59 from cell surfaces by activation of phospholipases, such as PI-PLC, may render neurons vulnerable to complement mediated attack, independent of upregulation of the *Cd59 *gene, as briefly described in the introduction [2,5,11,25].

CD59a^-/- ^mice which lack the *Cd59a *gene [16] provide an excellent *in vivo *model to test the relevance of the terminal complement pathway in contributing to secondary neuropathology after traumatic brain injury. Recent experimental studies revealed that CD59a^-/- ^mice are more susceptible to ischemia/reperfusion injuries [35] and to disease severity, myelin loss and axonal damage after EAE [15]. The latter study showed that areas of myelin loss and axonal damage in spinal cords of CD59a^-/- ^mice were associated with MAC deposition, implicating the complement MAC as a crucial mediator of neuropathology of this autoimmune disease [15]. The findings of exacerbated neurological impairment and increased neuronal cell loss in our current study on experimental closed head injury concur with the previous findings in the EAE model [15] and further underline the importance of the complement MAC in the pathophysiology of inflammatory central nervous system (CNS) disorders.

Traumatic brain injury is currently still lacking a specific pharmacological therapy designed to avoid induction of secondary brain injuries and delayed neuronal cell death [36]. However, in the field of therapeutic complement inhibitor development, there have been significant advances in recent years [37-40]. While some of these inhibitors have been successfully tested in experimental head injury models [24,25,41], the "bench-to-bedside" extrapolation to clinical applications in head injured patients has yet to be accomplished [36]. In this regard, the identification of human CD59 complement binding interfaces, as well as the recent development of human soluble mutant CD59-based compounds, which have been shown to exert an up to 3-fold increased complement inhibitory activity [42], may represent a promising future strategy for attenuating the terminal complement pathway-mediated neuropathology and the extent of secondary brain damage in head-injured patients.

## Competing interests

PFS is the co-inventor of a US patent filed (No. 11,441,828) entitled: *"Inhibition of the alternative complement pathway for treatment of traumatic brain injury, spinal cord injury, and related conditions*." The authors declare no other potential conflict of interest related to this project.

## Authors' contributions

PFS and BPM designed the study. BPM provided the CD59a^-/- ^mice and wild-type littermates. PFS and IL performed and supervised the animal experiments. MAF and BMT performed the ELISA experiments. IP, CS, CC, and IL performed the PCR and Western blot experiments. BPM, WRS, KB, OIS, and WE contributed to analysis and interpretation of the data and corrections of the manuscript. PFS and MAF wrote the first draft of the paper. All authors read and approved the final version of this manuscript.
